# Construction of networks with intrinsic temporal structure from UK cattle movement data

**DOI:** 10.1186/1746-6148-4-11

**Published:** 2008-03-20

**Authors:** M Fred Heath, Matthew C Vernon, Cerian R Webb

**Affiliations:** 1 University of Cambridge, Department of Veterinary Medicine, Madingley Road, Cambridge, UK; 2 University of Warwick, Department of Biological Sciences, Gibbet Hill Road, Coventry, UK

## Abstract

**Background:**

The implementation of national systems for recording the movements of cattle between agricultural holdings in the UK has enabled the development and parameterisation of network-based models for disease spread. These data can be used to form a network in which each cattle-holding location is represented by a single node and links between nodes are formed if there is a movement of cattle between them in the time period selected. However, this approach loses information on the time sequence of events thus reducing the accuracy of model predictions. In this paper, we propose an alternative way of structuring the data which retains information on the sequence of events but which still enables analysis of the structure of the network. The fundamental feature of this network is that nodes are not individual cattle-holding locations but are instead direct movements between pairs of locations. Links are made between nodes when the second node is a subsequent movement from the location that received the first movement.

**Results:**

Two networks are constructed assuming (i) a 7-day and (ii) a 14-day infectious period using British Cattle Movement Service (BCMS) data from 2004 and 2005. During this time period there were 4,183,670 movements that could be derived from the database. In both networks over 98% of the connected nodes formed a single giant weak component. Degree distributions show scale-free behaviour over a limited range only, due to the heterogeneity of locations: farms, markets, shows, abattoirs. Simulation of the spread of disease across the networks demonstrates that this approach to restructuring the data enables efficient comparison of the impact of transmission rates on disease spread.

**Conclusion:**

The redefinition of what constitutes a node has provided a means to simulate disease spread using all the information available in the BCMS database whilst providing a network that can be described analytically. This will enable the construction of generic networks with similar properties with which to assess the impact of small changes in  network structure on disease dynamics.

## Background

**Figure 1:**
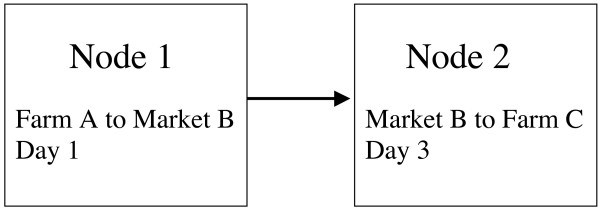
**Diagram of the structure of a link in the network**. An edge (link) will exist between a movement from Farm A to Market B (Node 1), and a movement from Market B to Farm C (Node 2), if Node 2 occurs on, or within a specified time limit after, the date of Node 1. Where the linking location is a Market, the time limit is 6 days.

**Figure 2:**
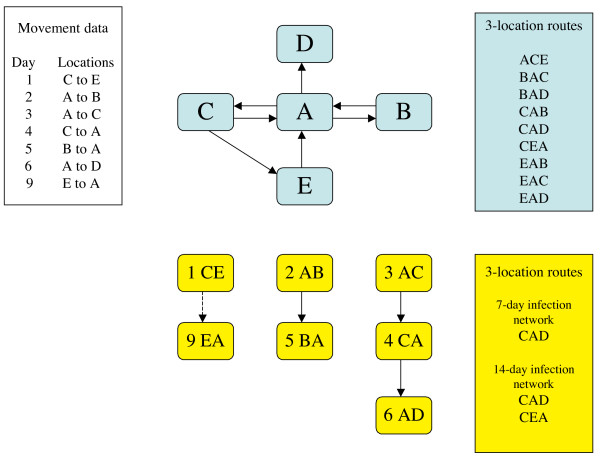
**Comparison of conventional and novel network structures for cattle movement data**. The "Movement data" box contains a fictional list of 7 dated cattle movements. These can be built into a conventional contact network (blue network) of 5 nodes and 7 edges. The networks built according to our novel procedure (see Text) are shown in the yellow diagram. There are 7 nodes. The 7-day infection network has 3 edges (the dashed edge is excluded by the time limit of 7 days between a movement on to location E and a movement off again, with location E being a farm). The 14-day infection network has 4 edges, inclusive of the dashed edge. The movement data defines contacts between pairs of locations; our procedure is designed to define meaningful routes of contact involving at least 3 locations. As can be seen from the coloured boxes, the conventional network provides 9 routes involving three distinct locations, whereas our networks show that only 1 (on the 7-day network) or at most 2 such routes are actually realistic routes for disease transmission.

In the basic SIR model for infectious disease [1], a population is divided into susceptible (S), infected (I) and resistant (R) sub-populations, and the spread of infectious disease in that population is calculated on the assumption of homogeneous mixing of the individuals, so that there is a fixed probability of contact between any two individuals. This model cannot be applied to disease spread between cattle farms, since they do not mix homogeneously and it is therefore essential to allow for the spatial distribution of farms, for example by controlling the probability of disease transmission between two farms by a function of their geographical proximity [2]. However, geographical proximity is not the only factor that may influence disease spread. Trade between farms creates a new topology in mapping disease risk. A more precise control of the probability of transmission could use the actual (and potentially infective) contacts between farms to build a contact network. Contact networks have been used in simulations of human disease (see review [3]), with the contact between two individuals being taken as persistent. For UK cattle farms, contact data is available in the form of the cattle movement database provided by the British Cattle Movement Service (BCMS). Because trade links are sporadic, movements are not well modelled as persistent contacts [4], and the temporal pattern of movements has important implications for the spread of infectious diseases. For structural studies of the cattle contact networks, movements have been regarded as quasi-persistent by considering the contacts resulting from movements during a relatively short period (e.g. 4 weeks) to be persistent through that period ([4,5]). Such networks can yield information relevant to non-persistent networks if they are considered ergodic [5]. Disease simulations have been carried out with a true temporal component, using a replay of the actual movements over a defined period [5,6]. Here, we attempt to use the BCMS movement data to build a network that incorporates the temporal sequence of movements between cattle premises, so that the structural features of such a network can be determined, and so that disease simulations can be run with less computational effort. The fundamental feature of our networks is that the nodes are not cattle farms but are instead movements between cattle farms (in fact between any cattle-holding premises or "locations"). Thus nodes have three basic properties: the source location, the target location, and the date of the movement. For simplicity, the number of animals moved is not included in the node definition. Edges (links) between nodes only exist when the target in one node is the source, in another node, of a movement on the same or a later date (Fig. 1). A further refinement is to include the type of each location (farm, market, slaughterhouse, showground, other) and to limit the time that may elapse after the first movement before the second movement is no longer linked to it. The time limit depends on the type of the linking location. We used the BCMS data for the whole of 2004 and 2005. The time limits for links involving farms were 7 days ("7-day infection network") or 14 days ("14-day infection network") (Fig. 2).

## Results

### Networks for structural investigations

#### Nodes and edges

The total number of movements available as nodes for the networks was 4,183,670. A larger proportion of the available nodes became connected to at least one other node in the 14-day infection network (81% vs 74%). The 14-day infection network had 71,965,734 edges, 5% more edges than the 7-day infection network (but slightly lower density: 6.3 vs 7.2 × 10^-6^). Unless otherwise stated, the following results exclude nodes that are not connected to any other node.

#### Components

A weak component of a network with directed edges (as we construct here) is a part of the network in which each node is connected to all the other nodes, along paths of edges *ignoring *the direction of the edges [7,8]. A strong component in such a network is similar, but with the direction of the edges *respected *[7,8]. The 7-day infection network has 17,110 weak components of 2 nodes, but the frequency of components of a given size drops off exponentially with increasing size and the largest minor weak component has 49 nodes. In this network, 97.9% of the connected nodes are actually in the giant weak component (3,022,611 nodes). The 14-day infection network has 12,184 weak components of 2 nodes, the largest minor weak component is 63 nodes, and 98.5% of the connected nodes are in the giant weak component (3,339,466 nodes). The directed structure of the networks makes strong components uninteresting. The theoretical maximum size of a strong component in a network of this kind is 2.

#### In- and out-degree

**Figure 3:**
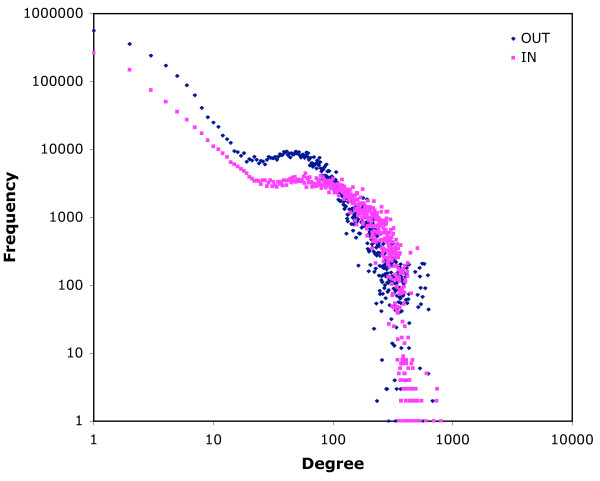
**In- and out-degree frequency distributions for the 7-day infection network**. Data for 2004 and 2005 for movements as nodes according to the 7-day infection network structure (see text).

**Figure 4:**
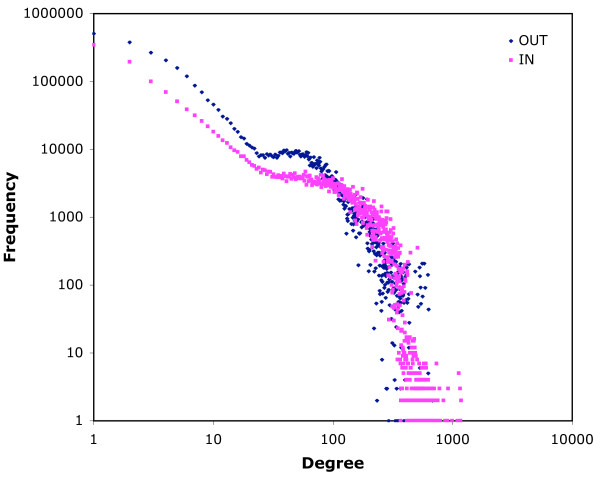
**In- and out-degree frequency distributions for the 14-day infection network**. Data for 2004 and 2005 for movements as nodes according to the 14-day infection network structure (see text).

**Figure 5:**
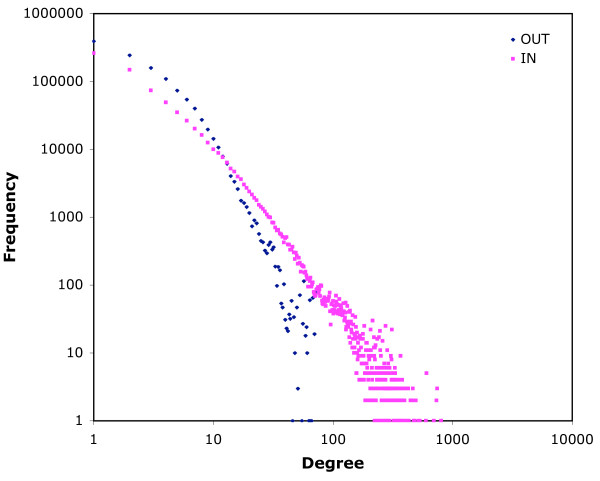
**In- and out-degree frequency distributions for farm movements only in the 7-day infection network**. Data as Fig 2, but including only edges where the linking location is a farm.

**Figure 6:**
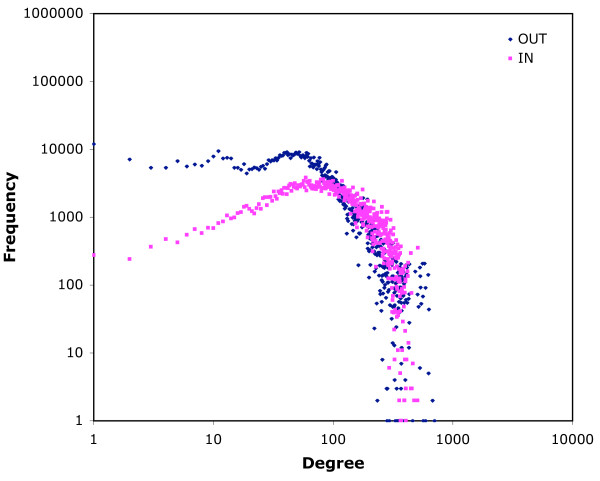
**In- and out-degree frequency distributions for market movements only in the 7-day infection network**. Data as Fig 2, but including only edges where the linking location is a market.

The maximum out-degree is the same for both networks (704), while the maximum in-degree is higher for the 14-day infection network (1,177 vs 804). The distributions of in-degree frequencies for both networks exhibit regions that correspond to scale-free networks, but the regions are small (Figs. 3 and 4). For low in-degrees (below 20) the plot shows a scale-free profile which then flattens out to a characteristic frequency of approximately 3000 up to in-degree 100. This is followed by an abrupt fall in frequency of nodes with high in-degree. Out-degree plots are similar, but more curvilinear in the first region (Figs. 3 and 4). Some of the anomalies are the result of the different behaviour of different types of locations. When in-degree is plotted only for movements from farms (so including only edges that link movements on and off the same farm), and out-degree for movements to farms (similarly), then more typically scale-free distributions are seen (7-day infection network, Fig. 5). Similar frequency plots for movements from and to markets only (for example) are very unlike scale-free distributions (7-day infection network, Fig. 6). Two-dimensional degree distributions reveal that very few nodes have both a high in- and out-degree, and that most have a low value for both (Table 1).

**Table 1:** Two-dimensional distribution of in- and out-degrees

**7-day infection network**		
	In-degree ≤ 10	In-degree > 10
Out-degree ≤ 10	54.1	18.6
Out-degree > 10	26.4	0.8
**14-day infection network**		
	In-degree ≤ 10	In-degree > 10
Out-degree ≤ 10	53.3	18.2
Out-degree > 10	26.7	1.8

Percentage of nodes in each network having the specified in- and out-degrees.

#### Dyad and triad censuses

**Figure 7:**
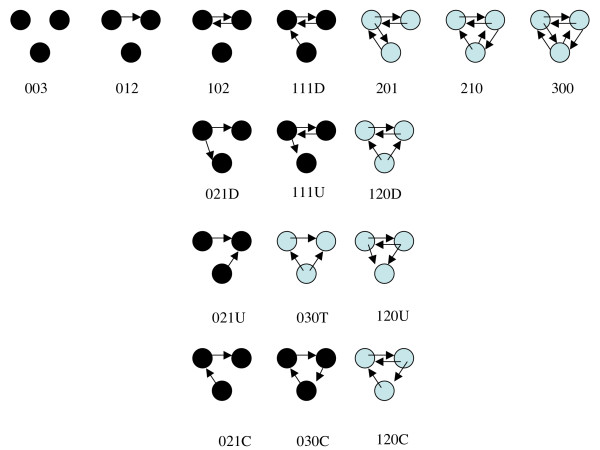
**The sixteen triad classes for the triad census**. Triads that are not allowed by the logic of the networks generated here, are shown with pale blue nodes.

Mutual dyads can only occur when reciprocal movements between two locations are made on the *same *day. Such events are rare, but logically occur in equal numbers in the two networks. The reciprocity of both the 7-day infection and 14-day infection networks is low (11.7 and 11.1 × 10^-4^, respectively). Similarly, in the triad census [9], 102 and 030C triads (see Fig. 7 for the structures of triad classes) are same-day events and have the same count in both networks. 030C triads are rare, and are the only triangles found, so the clustering coefficients are low (4.6 and 4.4 × 10^-6^, respectively). It is noteworthy that the triads 030T, 201, 120D, 120U, 120C, 210 and 300 are all forbidden by the rules for connecting nodes, and all yield zero counts in the partial triad censuses. These triads are shown with pale blue nodes in Fig. 7.

#### Disease simulations

As described in Methods, for each of the networks, 1000 disease simulations were run for each value of the parameter, q, which represents the probability of transmission of infection along an edge of the network from an infected node. These simulations were run, starting from a randomly-selected node, for 100 steps. The range and median of time spans represented by 100 steps is shown below for each network. The results of varying q on each network are given in Tables 2 and 3 as the peak number of infected movements, the maximum number of movements infected within a simulation, and the maximum number of steps before extinction of the infection (or percentage of simulations unextinguished by 100 steps), each collected over 1000 simulations.

**Table 2:** Disease simulation on the 7-day infection network

q	Max peak infected	Max cumulative	Max steps or final %
0.10	76	233	28
0.11	173	516	25
0.12	226	2,053	52
0.15	1,579	12,304	56
0.20	7,161	84,976	2.1%
0.50	23,550	533,241	16.5%
1.00	38,472	843,621	15.8%

*Max peak *infected is the peak number of infected movements on any one step of a simulation; *Max cumulative *is the maximum cumulative number of movements infected within a simulation; *Max steps or final % *is the maximum number of steps before extinction of the infection (or percentage of simulations unextinguished by 100 steps). Each is the maximum over 1000 simulations.

**Table 3:** Disease simulation on the 14-day infection network

q	Max peak infected	Max cumulative	Max steps or final %
0.10	582	3,620	49
0.11	914	7,916	71
0.12	2,534	21,105	99
0.15	11,201	146,380	3.8%
0.20	20,322	401,479	3.5%
0.50	60,362	973,599	95
1.00	81,323	1,275,646	76

*Max peak *infected is the peak number of infected movements on any one step of a simulation; *Max cumulative *is the maximum cumulative number of movements infected within a simulation; *Max steps or final % *is the maximum number of steps before extinction of the infection (or percentage of simulations unextinguished by 100 steps). Each is the maximum over 1000 simulations.

#### 7-day infection network

**Figure 8:**
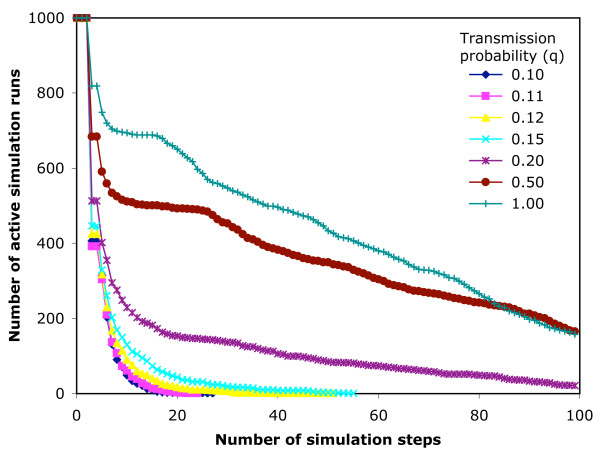
**The persistence of epidemic simulations on the 7-day infection network**. An SIR epidemic model is run 1000 times from random start nodes on the 7-day infection network structure, and the number of simulations not yet extinguished is plotted against the step number reached in the simulation.

**Figure 9:**
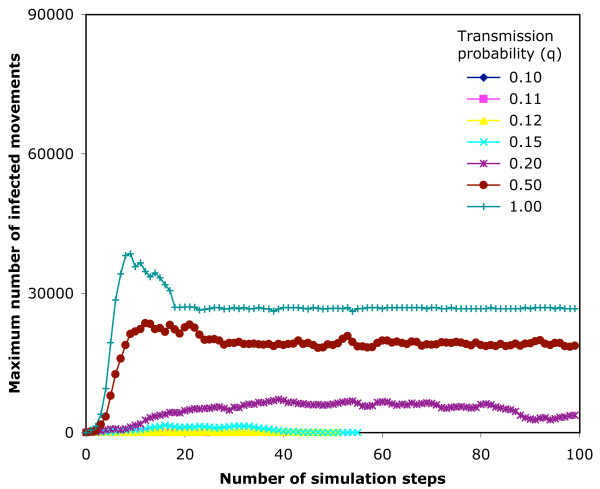
**Maximum sizes of epidemic simulations on the 7-day infection network**. An SIR epidemic model is run 1000 times from random start nodes on the 7-day infection network structure, and maximum number of currently infected nodes (for any of the simulations) at that step is plotted against the step number reached in the simulation.

In the 7-day infection network, 100 steps of simulation represents from 99 to187 days (median 140) of real time. There are 3,086,787 movements available to infect. The output of the simulations is shown in Table 2. The cumulative maximum for q = 1 represents 27.3% of available nodes. Fig. 8 shows the rate of extinction of simulations for the 7-day infection network, and Fig. 9 shows the maximum size of the epidemic at each step (i.e. the highest number of currently infected movements at that step in any of the 1000 simulations).

#### 14-day infection network

**Figure 10:**
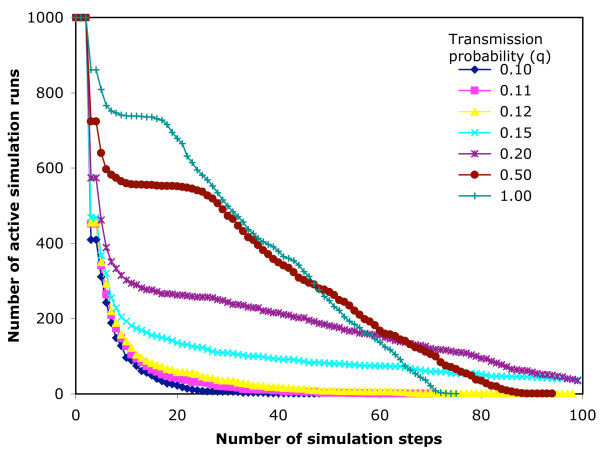
**The persistence of epidemic simulations on the 14-day infection network**. An SIR epidemic model is run 1000 times from random start nodes on the 14-day infection network structure, and the number of simulations not yet extinguished is plotted against the step number reached in the simulation.

**Figure 11:**
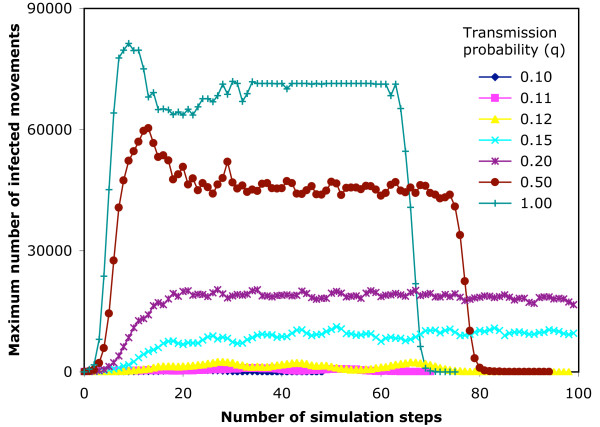
**Maximum sizes of epidemic simulations on the 14-day infection network**. An SIR epidemic model is run 1000 times from random start nodes on the 14-day infection network structure, and maximum number of currently infected nodes (for any of the simulations) at that step is plotted against the step number reached in the simulation.

In the 14-day infection network, 100 steps of simulation represents from 104 to 273 days (median 190) of real time. There are 3,391,471 movements available to infect. The output of the simulations is shown in Table 3. The cumulative maximum for q = 1 represents 37.6% of available nodes. Fig. 10 shows the rate of extinction of simulations for the 14-day infection network, and Fig. 11 shows the maximum size (over 1000 simulations) of the epidemic at each step.

## Discussion

While contact networks were a considerable step forward in the spatial modelling of disease spread, a simple structure of locations connected by the historical existence of cattle movements is not a good model of the temporal structure of these movements. We have viewed the movement data from a different perspective, using the movements as the nodes, which incorporate spatial relationships, and connecting them according to temporal criteria.

Our approach could be described as a heavily modified line-graph. A line-graph is a graph derived from an original network graph in the following way: each edge in the original graph becomes a node of the line-graph, and where two edges in the original graph share a node in common, their corresponding nodes in the line-graph are connected by an edge [7]. If our networks are regarded as derived from some notional static networks of locations and the movements between them, then they are line-graphs rendered much less dense by the application of our temporal sequencing rules, which use information not present in the notional static networks. We have found that the line-graph concept has been used before in epidemiological network models [10,11]. In that work, on human sexually-transmitted diseases (STDs), the static network consisted of sexually-active individuals as nodes with sexual relationships as undirected edges. As part of the calculations on the concurrency of such contacts, a full line-graph was constructed. All STD simulations were run on the static network, with some parameters derived from the line-graph. In our case, the static network is purely notional, the edges within it would be directed, and the derived line-graph would be heavily edited on the basis of temporal data. We then run our disease simulations on this derived network. Our approach might be beneficially used on the STD model.

There are concerns about the appropriateness of the BCMS movement data as the basis of a contact network. Doubts have been expressed about the accuracy and completeness of the records [12,13], and it is clear that some categories of movement are not recorded at all [6,13]. It is also necessary to consider other forms of contact between cattle farms that carry the risk of transmitting infection, such as vehicle movements and shared equipment [4]. In one recent study, these factors not involving cattle movements were found to be a minor component of a full contact network, whereas unrecorded movements on and off shared grazing were more important (M.C. Vernon, unpublished). We have analysed the BCMS data, but a similar analysis could be carried out with any contact list, for example one expanded from BCMS data to include other movements and other forms of contact.

The time slice of BCMS data analysed here (1 January 2004 – 31 December 2005) was chosen as the largest stable and coherent full calendar year segment from the data available to us. Earlier data was more fragmentary and was distorted by the FMD outbreak in 2001. The data for 2006 was incomplete. We chose to limit the length of time that an infected location remained infectious, and used the data on lengths of stays to determine appropriate limits for all-in, all-out operations. For farms, we selected limits within the period that an infection might remain undetected, and chose 7 and 14 days as these corresponded to peaks in the frequency of interval lengths between movements on and off farms presumably reflecting the weekly periodicity of farming practice or at least of reporting. These interval lengths are also of sufficient duration to be beyond the effects of the 6-day stand-still rule for UK cattle movements.

The 7-day and 14-day infection networks contain a high proportion of the available nodes (74 and 81%), with just over 98% of these connected nodes in a single giant weak component, in both networks. The method of construction of the networks means that the maximum size for a strong component is only 2 nodes. It also means that mutual dyads and triangles are uncommon (hence low reciprocities and clustering coefficients), and that 7 of the 16 triad classes are missing altogether. All these expectations are confirmed by the analyses. The networks, therefore, consist principally of asymmetric edges, all directed down a time line. The densities are low, as might be expected on a large network. The average in – (or out-) degree for the nodes in 7- and 14-day infection networks are 22 and 21, respectively.

The log-log plots of frequency of in-degree (Figs. 3 and 4) show only small areas of scale-free behaviour, a type of network structure that has been of particular interest to epidemiologists because infections can spread on a scale-free network without an epidemic threshold [14-17]. Out-degree plots (Figs. 3 and 4) show no scale-free regions. The patterns seen in Figs. 3 and 4 reflect the heterogeneity of the cattle-holding location types in the data used to construct the networks. The low degree region is mostly populated by movements off farms (when counting in-degree) and on to farms (when counting out-degree), as is shown for the 7-day infection network in Fig. 5. These movements show more classically scale-free behaviour. For movements that (in the same way) involve markets, however, the plots do not correspond to the scale-free profile (Fig. 6), but show a characteristic frequency in the region corresponding to the region 2 of Figs. 3 and 4, and an abrupt fall in frequency in the third region. What these Figures show in common is a marked heterogeneity of both in- and out-degree, which has been demonstrated, on undirected networks at least, to correspond to very rapid spreading of simulated epidemics [18].

The structural features determined for these networks, such as dyad and triad censuses and 2-dimensional degree distributions, could form the basic data for algorithms to produce generic networks representing UK cattle movements. Generic networks may be constructed such that the resulting network fits a set of structural criteria. For example, large networks may be rapidly constructed based on a particular two-dimensional degree distribution and dyad census, using a shuffling and re-wiring process [19].

For the disease simulations on the 7-day and 14-day infection networks, a run of 100 steps was used, as this gave effective time spans that covered several infectious periods (at least 14 and 7, respectively), but less than half the time span of the data, allowing the possibility that some epidemics will be completed before reaching the end of the data. The value of the transmission probability (q) is regarded as a sensitive parameter in disease simulation [6]. The q values chosen ran from 1.0, which would maximise the scope of the epidemic, down to 0.1, where the maximum epidemic size was less than 0.5% of the maximum for q = 1.0. For each set of conditions, 1000 simulations were run so that the maxima reported contain low frequency outcomes.

We found 3 possible outcomes from a simulation, as shown in Tables 2 and 3 and Figs. 8, 9, 10, 11, which depend on the probability of transmission of infection and the structure of the network. At low q, the epidemic extinguishes quickly (7-day, q ≤ 0.15; 14-day, q ≤ 0.12,). As q increases, the epidemic can maintain itself longer and is still not extinguished by the end of the data in a proportion of simulations (7-day, q ≥ 0.2; 14-day, 0.15 ≤ q ≤ 0.2). At higher q (≥ 0.5) in the 14-day infection network, the epidemic reaches the movements at the end of the time period captured by the network, and thus extinguishes abruptly. The maximum epidemic sizes for q = 1 were 27.3% and 37.6% of available nodes for the 7-day and 14-day infection networks, respectively. These results were obtained from arbitrary start points in the network. For smaller networks of this type, more efficient use of the available data might be achieved by selecting start nodes near the beginning of the time slice, with due allowance for differences in the movement pattern on different days of the week and in different seasons [20,21].

The simulation results are expressed in dynamic terms, as the object of study is the movement, not the holding, and we believe this provides a suitable way of viewing the vulnerability of the system. However, it might be of interest to know how the results translate into numbers of infected farms. The network structure we describe here does not allow the tracking of individual holdings during our disease simulations, which are only intended to be proof of concept. If such tracking were required, the adjacency list representing the network would have to be augmented with an extra parameter for each node pair to indicate the identity of the holding where the second movement terminated, and specialised software would have to be written to use this tracking information. With generic networks, rather than those directly derived from movement data, such tracking of holdings would not be possible. In the absence of such sophistications of network and software, we can suggest a rule of thumb for converting the number of infected movements into the number of infected farms. We start with the observation that 44% of the movements for 2004 and 2005 end at farms. In the case of the simulation on the 7-day infection network with probability of transmission (q) equal to 1, the maximum cumulative total of infected movements is (in round figures) 800,000 (Table 2), so 350,000 terminate at a farm. As 16 million farms were in use in 2004 plus 2005, it is conceivable that all these are "new" (i.e. not previously infected) farms, but knowledge of the industry and knowledge of the database, supported by a replay experiment, suggest that the proportion of new farms is much lower, perhaps 10–25%. The replay experiment uses a technique similar to one published for foot and mouth disease simulation [5,6], but independently derived (M.F. Heath, unpublished), in which the movements through the period are replayed in temporal sequence to follow the transmission of disease from an arbitrary infected holding. Using comparable conditions to those in the example under discussion (7-day infectious period; transmission probability = 1), the maximum cumulative total of infected farms was 50,000 (M.F. Heath, unpublished). Our rule of thumb, therefore, is that the maximum cumulative number of infected farms is 5–10% of the maximum cumulative total of infected movements. For our largest simulated "epidemic" (14-day infection network; q = 1 (Table 3)) this suggests up to 120,000 farms might be infected, whereas for our smallest (7-day infection network; q = 0.10 (Table 2)) the maximum might be as low as 10 farms.

The simulation results indicate that networks constructed in this way may be used to efficiently test disease transmission scenarios on the UK cattle industry, avoiding the need to develop dynamic network models.

## Conclusion

These networks incorporate spatial relationships, in common with other contact networks, but also incorporate temporal relationships. The structures include a giant weak component, and some unusual features resulting from the lack of strong components greater than 2 nodes. The variety of location types in the underlying location population leads to non-scale-free degree distributions. The simple simulations of the spread of infection on the networks indicate that there are typical behaviours, such as extinction of an epidemic at low infectivity but persistence at high infectivity. We conclude that networks built with movements as nodes could be used for analysis and for simulation to give a new insight into the spread of infection in the UK cattle herd.

## Methods

### Cattle movements

BCMS data was provided by DEFRA from the RADAR project on 24 May 2006, covering movements for the period from January 1999 to April 2006. This was approximately 21 GB of data. The data was loaded into an Oracle™ database (Oracle Corporation, Redwood Shores, California, USA). In this analysis we have selected all records for two complete years, 1 January 2004 to 31 December 2005.

The main table of BCMS data in RADAR consists of "stay on location" records for individual cattle. These include fields for the individual livestock (cow) identifier, the location identifier, the arrival date, the departure date, the type of movement on to the location (e.g. by birth, or by a normal trade transfer on to the location) and the type of movement off the location (e.g. death, normal trade transfer off). From this table may be derived a set of "movement" records. These are constructed from pairs of records relating to the same animal, where the departure date of the first record is equal to the arrival date for the second record. This will miss some movements where the arrival at the second location is on a later day than that of the departure from the first location. The logic of the data extraction would collect "movements" which were not directly from the first location to the second, but actually involved intermediate locations, if the rule requiring the dates to be the same were to be relaxed. The limit of precision of timing to one day means that sometimes two "stay on location" records may be started and completed within one day and their temporal sequence cannot be recovered. Some can be resolved by a further rule, that the type of movement off the first location may not be "DEATH". No "movement" record is constructed if either the location-from or the location-to is undefined (coded as "-1" in the RADAR data; 4.4% of all "stays on location"). All such locations would match in the next stage of data extraction, which would be misleading. The "movements" are collected from the whole of 2004 and 2005.

Each record consists of the location-from, the location-to and the date of the movement. All movements where one of the locations was unknown have been eliminated. Since we are interested in the cattle-holding locations rather than individual animals, the dataset is simplified by removing duplicate records, thus reducing the movement of a group of cattle between two locations to a single contact between those locations, irrespective of the number of cattle involved. The tie strength (i.e. the number of animals moved) is ignored for the purposes of this analysis.

There is a second BCMS data table that contains details of all the locations in the main table, including the location type, where known. The preponderant types are Agricultural Holding (64% of "stays on location" with known location), Slaughterhouse (Red Meat) (19%), Market (15%), Landless Keeper (1%) and Showground (0.3%). For 0.3% of stays with known location, the location type is unknown, but for the purposes of this analysis they are considered to be Agricultural Holdings. Each "movement" record receives a type-from record and a type-to record from the location table, based on its location-from and location-to fields, and finally a unique identity number. These records are the nodes of the network. The list of nodes can be used to generate networks for structural investigation and simulations of disease spread.

### Contact networks

The edges of a network are defined as directed links from a first movement to a second movement, where the location-to of the first movement is the same as the location-from of the second, and the second movement occurs on or after the date of the first movement. The network thus incorporates the temporal sequencing of the contacts between locations. We are concerned with the risk of disease transmission by these contacts. After a period of time, the risk that an infection, brought into a location by a contact, will be passed on in a subsequent contact, will have declined, so edges are not constructed that link two movements that are separated in time by a period greater than an arbitrary limit. If the number of days that elapse after the first movement exceeds the time limit, a link is no longer made (Fig. 2).

Two methods have been used to set time limits, one for locations where an all-in, all-out policy is expected and another for "Farms". For locations where an all-in, all-out policy is expected, this maximum time is set by inspection of the distribution of lengths of "stay on location" for these location types in the expectation that one group of animals will not mix with the next group. For Markets, Showgrounds, Slaughterhouses and Other non-Farm locations the maximum time is thus set at 6, 5, 5 and 4 days, respectively. For Agricultural Holdings and Landless Keepers (referred to here collectively as "Farms"), the maximum will depend on the nature of the disease that is to be modelled. We have chosen two illustrative values for this time limit, and therefore constructed two different networks from the data. The values used (for all Farms) were 7 days and 14 days.

The network is defined by an adjacency list consisting of pairs of numbers: the node identifier of the starting node of the edge and the node identifier of the ending node of the link. Thus the network will incorporate in a simple adjacency list the temporal sequencing of the contacts and the interaction between the properties of the type of disease being modelled and the types of locations on which the cattle are held. The construction of the network, as described above, ensures that all the links are real routes through which infection can pass from one location via an intermediary location to a third location. This is in contrast to networks where the nodes are locations and the edges are movements. These lose the temporal sequence information available in the source movement data.

For the two networks (with 7-day infectious period, denoted by "the 7-day infection network", and with 14-day infectious period, denoted by "the 14-day infection network", as described above), the following standard network parameters [9] have been calculated within Oracle, by processing Oracle output in MS Excel, and with our own routines (the Contagion library [19]):

1 Number of nodes in the network and the number of edges (links) between them.

2 Density, measured as the proportion of all the theoretically possible edges between nodes that actually exist. This tends to be small for large networks.

3 Out-degree frequency distribution. The out-degree of a node is the number of edges that start from that node.

4 In-degree frequency distribution. The in-degree of a node is the number of edges that end at that node.

5 Frequency distribution of in- versus out-degree per node ("2-dimensional" frequency distribution).

6 Dyad census, being the count of the types of connection between pairs of nodes and thus giving information on the structure of the network. All possible pairs of nodes in the network are considered, and are categorised as mutual (edges in both directions), asymmetric (only one edge between them) or null (unconnected) dyads. Uses our routine, dyad_census [19].

7 Reciprocity, calculated as the proportion of non-null dyads that are mutual, quantitates the reciprocal nature of the linkages.

8 Partial triad census. The triad census is the count of the types of connection between trios of nodes and thus gives information on the structure of the network. All possible trios of nodes in the network are considered, and are categorised into 16 classes in a manner analogous to dyads [8]. Our census is partial as it does not give a precise count of the null triads (unconnected trios). Uses our routine, triad_census [19].

9 Clustering coefficient, derived from the triad census [22] as the ratio of triangles (nodes linked to two other nodes, which are themselves interlinked) to triples (nodes linked to two other nodes). This quantitates the tendency of nodes to formed interlinked clusters.

10 Frequency distribution of sizes of weak components. These are sets of nodes that are linked together, but not necessarily linked directly or reciprocally. The sizes of these components reflect the overall reachability of any node from any other node. Uses our routine, weak_component_count [19].

### Disease simulations

In order to demonstrate in principle the simulation of the spread of disease on our networks, we applied the sir_net function [19], which was originally designed for the simulation of disease spread on a static network with locations as nodes and movements as directed (but not temporally sequenced) links. In the simulations described here:

1. The nodes are movements and the simulation begins with the random assignment of one movement as "infected". This effectively places infected cattle on the target location of the movement, so that the subsequent movements that start from that location are infected.

2. The edges are directed and run between movements under the criteria described for these networks above.

3. Each iteration tests the spread of infection along the edges from infected movements, so infection spreads in step that are not synchronised in time. The number of steps used in the simulations was 100. To assist in putting this in a context of time periods, the range of time intervals represented by 100 steps is presented for each network in the Results.

4. Each node remained infectious for only 1 step, as the edges leading from an infected movement should only be tested once.

5. The probability of transmission of infection along an edge (q) was constant for the simulation. To test for transmission along each edge at risk, a random positive number less than 1 was compared with q. Transmission occurred if its value was less than or equal to q.

6. The values of q, that were simulated on each of the networks, were 0.1, 0.11, 0.12, 0.15, 0.2, 0.5, 1.0.

7. 1000 simulations were run for each value of q, for each network.

## Authors' contributions

MFH proposed the concept, carried out the analysis and drafted the manuscript. MCV prepared the movement data, participated in the study design, advised on data extraction and analysis, and assisted in revising the manuscript. CRW participated in the study design and interpretation, and assisted in revising the manuscript. All the authors read and approved the final manuscript.
